# Evaluation of Clinical and Radiological Results of Humeral Diaphyseal Fractures with Treated Sarmiento Brace

**DOI:** 10.7759/cureus.7077

**Published:** 2020-02-22

**Authors:** Can Doruk Basa, Ismail Eralp Kacmaz, Anil Murat Ozturk, Levent Kucuk, Erhan Coskunol

**Affiliations:** 1 Orthopaedics, Tepecik Training and Research Hospital, Izmir, TUR; 2 Orthopaedics and Traumatology, Ege University, Faculty of Medicine, Izmir, TUR

**Keywords:** humerus fracture, sarmiento, functional brace

## Abstract

Aim

In our study, we aimed to evaluate the duration and rate of the union of adult humerus diaphysis fractures treated with a functional brace.

Methods

Forty-six adult patients admitted to our hospital with humeral diaphyseal fracture between January 2010 and April 2014 and treated with a functional brace were evaluated retrospectively. The demographic data, fracture type, level of fracture, and presence of bone union of the patients were evaluated from clinic records. The Disabilities of the Arm, Shoulder and Hand (Quick-DASH) questionnaire score of the patients was registered for patients and analyzed, and its correlation with parameters such as patient age and fracture bone union time was evaluated.

Results

It was observed that of the patients with the mean age of 45.5 years, six (13.6%) had non-union and five had delayed bone union (10.86%). Three (6.5%) patients had radial nerve injury, and all recovered without sequelae. Out of the patients with the bone union, 12 (30%) had an angulation above give degrees, and 3 (7.5%) had radiological shortness. The mean Quick-DASH score was 6.7, and there was no statistically significant correlation with parameters such as fracture type, level, angulation, radiological shortness and radial nerve involvement (p>0.05).

Conclusions

A functional brace is a good treatment choice with low complication rates and has satisfactory bone union rates in humerus diaphyseal fractures.

## Introduction

Humeral diaphyseal fractures are common fractures in all age groups and account for 3% of all long bone fractures and 5-8% of all fractures [1-3]. The incidence of humeral diaphyseal fractures is approximately 13 in 100,000 people per year [4].

In general, conservative treatments are tried in the treatment of humeral diaphyseal fractures, and successful results are achieved. Among the conservative treatments, functional brace techniques, which were defined and popularized by Sarmiento in 1977, are among the most preferred methods [5].

Although the rates of bone union with functional brace are variable in the literature, the review by Papasoulis et al. found that the mean rate was 94.5%, ranging from 77.4% to 100% [6]. There is no consensus in the literature regarding the mean bone union time, as well as bone union rates. However, the mean bone union time has been reported to be between 8-14 weeks in general [6]. In the literature, some fracture localizations are also thought to be facilitating in terms of non-union; however, there is no consensus on this view [7]. Koch et al. emphasized that non-union may arise in cases of transverse fractures, but Ring et al. stated that non-union was more common in cases of proximal spiral-oblique fractures [8, 9].

The aim of our study is to evaluate the results of the technique that we routinely use in our clinical practice in light of these data regarding humeral diaphyseal fractures in the literature and its correlation with the literature and to reveal similar and different aspects with the literature.

## Materials and methods

In our study, the outpatient clinic examination records and radiological data of 46 patients admitted to the emergency unit of our hospital with humeral diaphyseal fracture and treated with Sarmiento brace between January 2010 and April 2014 were retrospectively analyzed. The informed consent forms were obtained from all participants. Fractures at the distal of fossa olecrane and at the proximal of the surgical neck, patients with open fractures, those with pathological fractures, pediatric patients under the age of sixteen, and those with inadequate radiological and clinical data were not included in the study [10].

The demographic data of the patients, such as age, fracture area, the sex were recorded. The age groups of the patients were defined and grouped as young adults age between 16-40 years, middle age between 40-64 years, and advanced age over 64 years. The level of the fracture was divided into three regions as proximal, middle and distal diaphyseal fractures. The fracture type was evaluated according to the Association for Osteosynthesis/Association for the Study of Internal Fixation (AO/ASIF) classification, and the fracture pattern was divided into transverse, oblique, spiral, or segmental butterfly fragment (fracture) groups [11]. The patients were evaluated for the presence of radial nerve involvement.

The injured extremities were initially stabilized in an above-the-elbow cast or a coaptation splint for an average of seven days (3-10) prior to the application of the prefabricated brace. The type of first stabilization technique was noted. The bracing was performed by the same technician, as described by Sarmiento [5]. In cases with distal humerus fractures, the braces were modified with the application of a device that went beyond the elbow. All braces and control X-rays were evaluated by the senior author. After first control, X-rays reduction aid pads have been used in required cases in order to provide acceptable alignment after bracing. Radiographs were made at each follow-up visit until the fracture healed. A static radial palsy brace for a hand was used for the patients with radial nerve involvement.

The patients were followed up with standard treatment and follow-up protocol and by a single physician, and after bracing rehabilitation started immediately. The elbow movement started immediately, except in cases with distal fractures, after proper alignment was obtained. In cases with distal fractures, the application of brace was removed, and the elbow was freed to gain elbow movements after the bone union has started.

Bone union was evaluated according to the presence of callus on bilateral (A-P) humerus X-rays and the absence of local sensitivity in the fracture line of the patient. Unions over four months after trauma were named delayed unions [12].

After fracture union was completed, angulation and presence of shortness on direct X-rays were evaluated. The direction of angulation was not evaluated in our study. The patients with an angulation of 5° and below were considered as no angulation, whereas angulations above 5° were considered as angulation.

The Quick-DASH scoring adapted to Turkish society from the Disability of the Arm, Shoulder and The Hand (DASH) scoring that questions level of pain and level of daily activity that would require the use of a hand was administered to the patients [12]. The Quick-DASH scoring is a scoring system that aims to show the use of the upper limb in daily activities with a score from one to five; as the score increases, the result gets worse. The Quick-DASH scores of the patients were found to be filled after completion of bone union or in patients with non-union in long-term follow-up (more than eight months).

The SPSS 22.0 software (IBM Corporation, Armonk, USA) was used to analyze the data. The independent-samples t-test was used with the bootstrap results to compare two independent groups, while the Mann-Whitney U test and the Kruskal-Wallis H test were used with the Monte Carlo simulation technique. Spearman’s rho test was used to analyze the correlations of the variables with each other. The Pearson chi-square and Fisher's exact tests were tested by the Monte Carlo simulation technique for the comparison of the categorical data. The data were analyzed at a confidence level of 95%, and the p-value was accepted as lower than 0.05.

## Results

The demographic data of the patients in our study showed equal female-male distribution. The mean follow-up duration of the patients was 35.1 ± 18.05 months (range: 11-63 months). When the age groups of the patients were evaluated, it was seen that there were twenty patients in the young adult group, sixteen in the middle age group, and ten in the advanced age group. The mean age of the patients was 45.5 ± 21.5 years. The patients' fracture side, fracture type, fracture level, and distribution of types according to the AO/ASIF classification are presented in Table 1.

**Table 1:** Fracture side, type, level of the patients and evaluation according to the AO/ASIF classification AO/ASIF - Association for Osteosynthesis/Association for the Study of Internal Fixation

		N (%)
Side	Right	20 (43.5)
	Left	26 (56.5)
Fracture level	Proximal	11 (23.9)
	Moderate	23 (50)
	Distal	12 (26.1)
Fracture type	Transverse	9 (19.6)
	Oblique	8 (17.4)
	Spiral	26 (56.5)
	Segmental butterfly fragment	3 (6.5)
Type according to the AO/ASIF classification	Type A	43 (93.5)
	Type B	3 (6.5)

For the first interventions of the patients at the emergency unit, U splint, hanging cast, Velpeau bandage, or long arm splint with shoulder support were used. The decision of the first intervention was taken depending on fracture type and level. In the proximal third fracture, Velpeau bandage or long arm splint were used. In distal and mid-third fractures, U splint or hanging cast were used. In our study, it was observed that the most common technique used was shoulder-supported long arm splint (23 patients); the remaining patients had their first intervention with other techniques.

Three (6.5%) patients had radial nerve involvement. One of these patients had mid-diaphyseal type A1 fracture, one had mid-diaphyseal A2 type fracture, and one had proximal diaphyseal A1 type fracture. The radial nerve healed totally in four months in the first patient and in six months in the other two patients.

Of the forty-six patients, six (13.04%) had nonunion. It was found that the remaining forty patients (10.86%) had a bone union time of over four months, which was characterized as delayed union, and 35 patients' fracture union was completed in appropriate time (mean: 2.9 ± 1.53 months, range: 1-6 months). Five out of patients with nonunion fraction were operated and the mean duration until the operation was 6.2 ± 2.17 months (range: 3-8 months). One patient could not undergo an operation due to comorbidities. The postoperative union status and functions of the patients were not evaluated. One patient in our study was found to have a periprosthetic fracture, a spiral oblique fracture, and a union.

**Figure 1:**
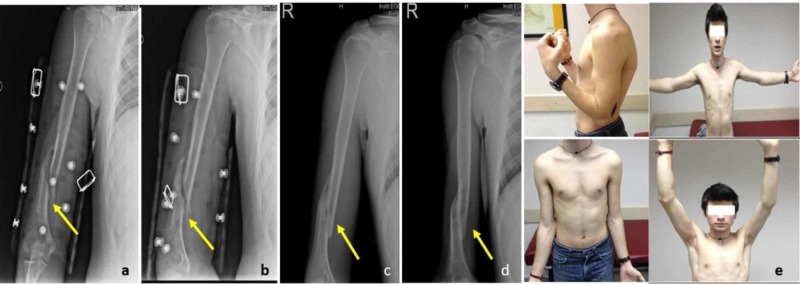
Radiographic results A-B: Radiographic result on month one C-D: Radiographic result on month four E: Clinical outcome on month four; the range of motions of elbow and shoulders are the same with uninjured extremity

When bilateral direct X-rays of the patients with union taken during the last follow-up were evaluated, 28 (70%) of the patients had no angulation, 12 (30%) had angulation. Only three (7.5%) patients had shortness. Radiographic and clinical images of one patient are shown in Figure 1. The correlation of fracture union with patient age, fracture level, and fracture type were investigated, and no statistically significant result was obtained (Table 2).

**Table 2:** Evaluation of correlation between union status and fracture type, level and patient age (significance level p<0.05)

		Union status		p-value
		No (n=6)	Yes (n=40)	
Level	Proximal	3 (50%)	8 (20%)	0.334
	Moderate	2 (33.3%)	21 (52.5%)	
	Distal	1 (16.7%)	11 (27.5%)	
Fracture type	Transverse	1 (16.7%)	8 (20%)	0.130
	Oblique	3 (50%)	5 (12,5%)	
	Spiral	2 (33.3%)	24 (60%)	
	Segmental butterfly fragment	0	3 (7.5%)	
Age		59.3 ± 20.04	43.4 ± 21.17	0.06

The mean DASH score of the patients was found to be 6.7 ± 10.53 (range: 0-47.7). There was no statistically significant correlation between fracture level, fracture type, radial nerve involvement, fracture angulation and fracture shortness, and Quick-DASH scoring (p > 0.05).

**Figure 2:**
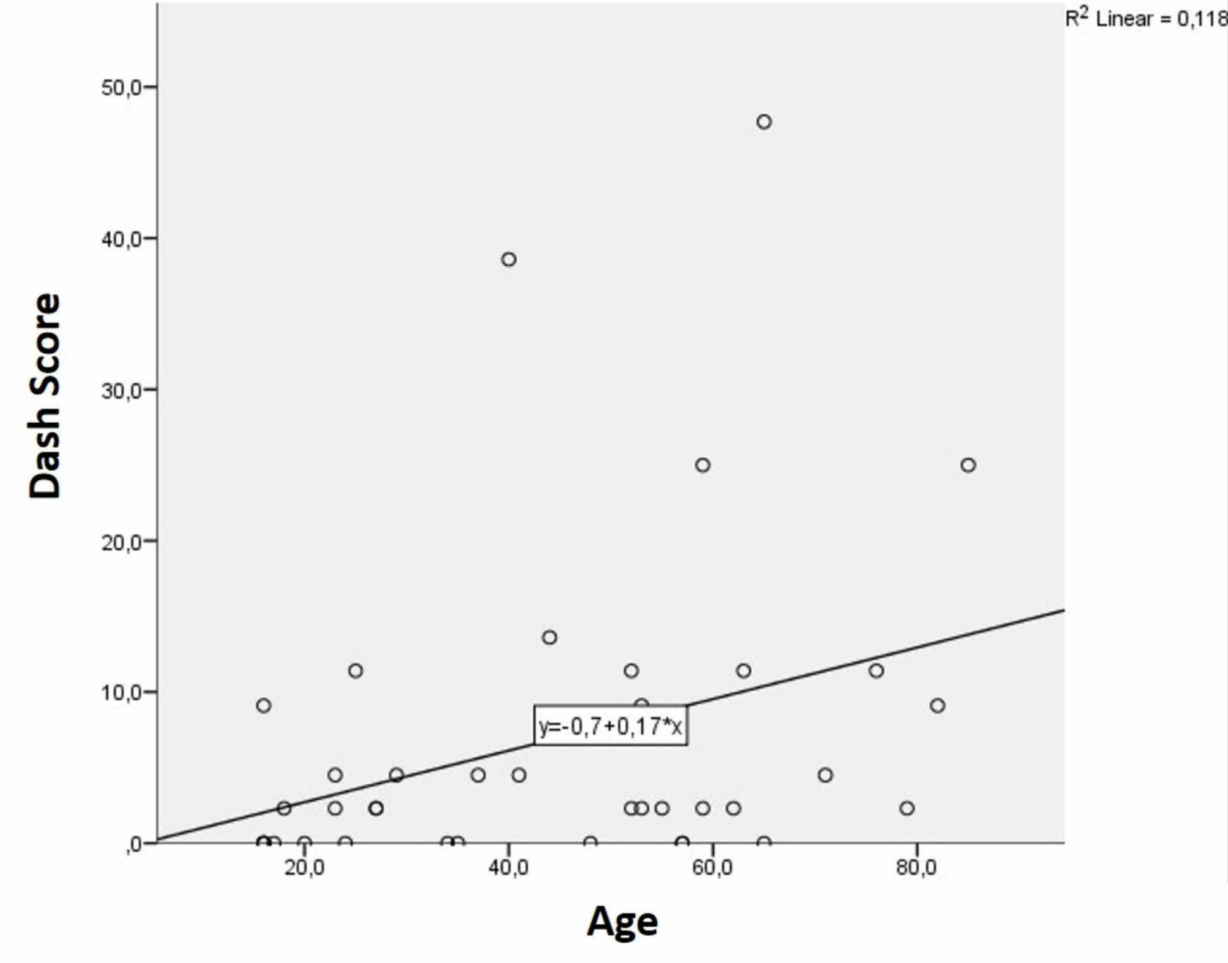
Evaluation of correlation between patient age and Quick-DASH score DASH - The Disabilities of the Arm, Shoulder and Hand

**Figure 3:**
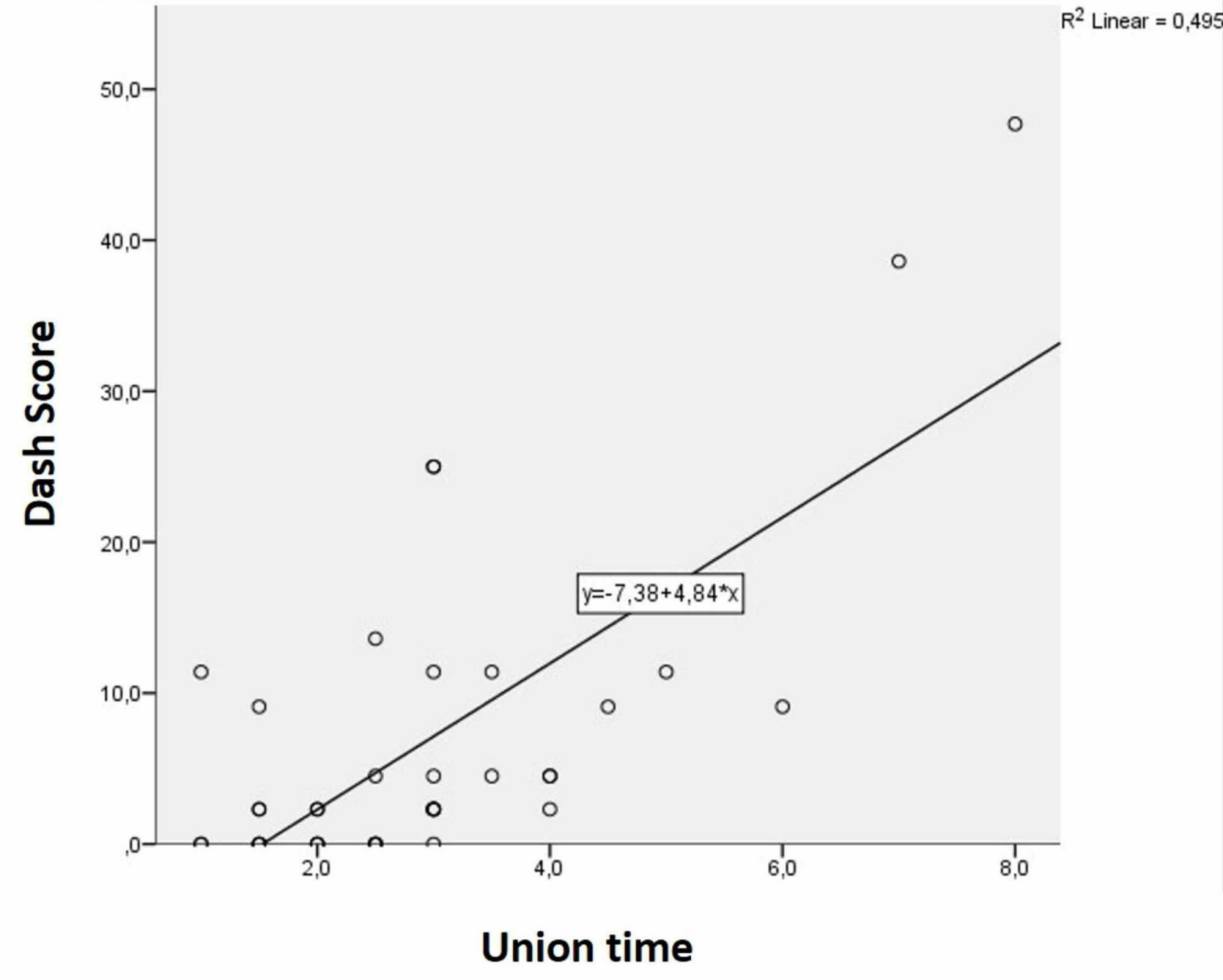
Evaluation of correlation between union time and Quick-DASH score DASH - The Disabilities of the Arm, Shoulder and Hand

In the correlation analysis (between the Quick-DASH scores, age and union time), the correlation coefficient between 0.10-0.29 was interpreted as low correlation, 0.30-0.49, as a moderate correlation and 0.50-1 as high correlation. There was a moderate positive correlation between age and Quick-DASH, which was statistically significant (r=0.422; p=0.007) (Figure 2). There was a high positive correlation between union time and Quick-DASH score (r=0.575), which was also found to be statistically significant (p<0.001) (Figure 3). There was also a moderate positive correlation (r=0.476) between age and union time of the patients, which was also statistically significant (p=0.002). The correlation analyses are summarized in Table 3. 

**Table 3:** Evaluation of correlation between age, union time and Quick DASH score with the Spearman's rho test sig - significance DASH - The Disabilities of the Arm, Shoulder and Hand

			Age	Union time	Quick-DASH score
Spearman's rho	Age	Correlation coefficient	1.000	0.476	0.422
		Sig. (2-tailed)		0.002	0.007
		N	46	40	40
	Union time	Correlation coefficient	0.476	1.000	0.575
		Sig. (2-tailed)	0.002		0.000
		N	40	40	40
	Quick-DASH score	Correlation coefficient	0.422	0.575	1.000
		Sig. (2-tailed)	0.007	0.000	

## Discussion

The standard gold treatment in cases of humeral diaphyseal fracture seen in the adult age group is considered to be conservative treatment. In a systematic review by Updegrove et al. on humeral shaft fractures, this view has been supported; however, they thought that a specific approach would be appropriate considering the fracture type, level, and patient-specific risk factors [13]. High nonunion rates in spiral oblique-type humeral shaft fractures of the proximal region mentioned in this review are thought to be higher in the studies by Ali et al., Rutgers et al., Toivanen et al., which are in particular considered to be caused by deforming-effect of the pectoralis major and deltoid muscle [7, 14, 15]. When the patients with nonunion were analyzed in our study, it was found that the nonunion rates in the proximal region and spiral fractures were higher, and the results were similar to the literature. It should be kept in mind that nonunion may arise more commonly in cases of spiral oblique-type proximal diaphyseal fractures.

The indications may also vary depending on the types of fractures. In particular, as the rate of shoulder and elbow arthroplasty increased, the incidence rate of periprosthetic fractures increased [16]. The most appropriate treatment for periprosthetic fractures has not yet been established. However, conservative treatment can be administered, if the alignment is suitable and the prosthesis is stable, but if the prosthesis is not stable, it has been accepted to carry out revision surgery [17]. In our study, there was only one patient with a periprosthetic fracture, and successful results were obtained with conservative treatment.

In our study, it was observed that radiological angulation over five degrees in patients with union occurred in 12 patients (30%), while radiological shortness arose in three patients (7.5%). However, these were not associated with Quick-DASH scoring, which assesses patient outcomes. Considering the literature, a study by Shields et al. investigating the residual radiological angulation and shortness, concluded that the residual deformities of the sagittal and coronal plane were not correlated with the functional outcome [18]. These results are similar to that of our study.

The incidence rate of radial nerve injury in cases of humeral closed shaft fracture has been reported to be up to 18% [19]. As a general acceptance, it is recommended to follow-up without performing exploration in cases of humeral shaft fracture, if the radial nerve injury has not occurred after fracture manipulation [20]. In our study, radial nerve injury was found in three patients (6.5%). The reverse of all these injuries was evaluated to be similar to the literature.

When the results of our study are analyzed, we see a positive correlation between age and Quick-DASH score and union time. Similar results were also found in the study by Harkin et al. investigated all humeral fractures in a large series [21]. It was observed that the patients followed up conservatively, four had nonunion, which was due to comorbidities and additional surgical risks in the elderly patient population. In addition, Wang et al. also analyzed humeral fractures in the elderly patient group and demonstrated that humeral fractures resulted in severe functional loss, even mortality [22]. The fact that the Quick-DASH score in our study correlated with age was similar to this study.

Plate osteosynthesis, intramedullary rod or external fixator are also among the surgical techniques that can be used in humeral fractures [23]. In their review, Walker et al. emphasized that conservative treatment might provide good results, and that union rates were similar, while the complication rates were higher in all surgical treatments. Knowing the complication rates in treatment choice should also be kept in mind when choosing treatment [23, 24].

Our study has some weaknesses. The most important of these is the absence of a control group and the fact that conservative patient selection is not randomized. The strengths of our study are that the follow-up was carried out by a single physician, and also, functional braces were administered by a single technician. In this way, it has been tried to provide standardization. Other strengths of our study can be considered as having a large series comparable to the literature and evaluating patient functions as well as the radiological union.

## Conclusions

In this study, we have evaluated the clinical and functional results of humeral shaft fractures who had conservative treatment retrospectively. The functional brace is one of the treatment techniques that can be used safely in adult humeral shaft fractures with high union rates, good functional results, and low complication rates.
